# Contribution of gap junctional communication between tumor cells and astroglia to the invasion of the brain parenchyma by human glioblastomas

**DOI:** 10.1186/1471-2121-6-7

**Published:** 2005-02-16

**Authors:** Roxane Oliveira, Christo Christov, Jean Sébastien Guillamo, Sophie de Boüard, Stéphane Palfi, Laurent Venance, Marcienne Tardy, Marc Peschanski

**Affiliations:** 1 INSERM/UPVM 421, Plasticité cellulaire et thérapeutique, Faculté de Médecine, 94010 Créteil cedex France; 2 Service de neurochirurgie, CHU Henri Mondor, 94010 Créteil cedex France; 3 INSERM U 114, NeuroBiologie, Collège de France, Place Marcellin Berthelot, 75005 Paris cedex France

## Abstract

**Background:**

Gliomas are "intraparenchymally metastatic" tumors, invading the brain in a non-destructive way that suggests cooperation between glioma cells and their environment. Recent studies using an engineered rodent C6 tumor cell line have pointed to mechanisms of invasion that involved gap junctional communication (GJC), with connexin 43 as a substrate. We explored whether this concept may have clinical relevance by analyzing the participation of GJC in human glioblastoma invasion.

**Results:**

Three complementary in vitro assays were used: (i) seeding on collagen IV, to analyze homocellular interactions between tumor cells (ii) co-cultures with astrocytes, to study glioblastoma/astrocytes relationships and (iii) implantation into organotypic brain slice cultures, that mimic the three-dimensional parenchymal environment. Carbenoxolone, a potent blocker of GJC, inhibited cell migration in the two latter models. It paradoxically increased it in the first one. These results showed that homocellular interaction between tumor cells supports intercellular adhesion, whereas heterocellular glioblastoma/astrocytes interactions through functional GJC conversely support tumor cell migration. As demonstrated for the rodent cell line, connexin 43 may be responsible for this heterocellular functional coupling. Its levels of expression, high in astrocytes, correlated positively with invasiveness in biopsied tumors.

**Conclusions:**

our results underscore the potential clinical relevance of the concept put forward by other authors based on experiments with a rodent cell line, that glioblastoma cells use astrocytes as a substrate for their migration by subverting communication through connexin 43-dependent gap junctions.

## Background

Glioblastoma, the most aggressive primary brain tumor, is invariably associated with profuse and long-distance invasion of the brain parenchyma. Accordingly, this tumor has been defined as "intraparenchymally metastatic" [1]. Intra-parenchymal dissemination is largely responsible for systematic recurrence despite treatments [2], and poor prognosis with a median survival time not exceeding one year [3]. Preferential routes of long-distance dissemination have been identified, among which the basal lamina of blood vessels and fiber tracts [1,4]. Numerous studies have revealed mechanisms of invasion in those conditions, specifying the molecular bases of glioma cells interaction with specific ligands of the brain extracellular matrix, their proteolytic modification by glioma cells, and the corresponding cellular receptors [2,5]. Less is known about the mechanisms that underlie the migration of glioma cells among neural cells, in the brain parenchyma, despite its profuseness in the area that surrounds the tumor mass, and its major role in recurrence.

We have undertaken to identify the mechanisms of this particular type of glioma invasion, on the basis of the hypothesis that cells in the brain parenchyma may play a supportive role in this process. One key for the elaboration of this working hypothesis has been the demonstration that this intra-parenchymal invasion occurs in a non-destructive way (see refs and discussion in Bernstein, 1996), as this suggests that invading human glioblastoma cells may establish relationships of cooperation with their environment and in particular with resident brain cells. We have focused our attention on gap junctions because of two complementary reasons. First, evidence from peripheral tumors such as melanoma has implicated gap junctions in tumor cell migration [6]. Second, gap-junctional communication via connexin 43 (Cx43) between astrocytes and glioma cells has been demonstrated [7], and linked to a phenotypic transformation of astrocyte which may render the brain parenchyma permissive to glioma invasion. In a recent study, Lin et al. have strongly supported this hypothesis by establishing that non-migratory rodent C6 tumor cells display a migratory behavior following transplantation into the rat brain, when engineered to synthesize Cx43 [8]. Although Cx43 was shown to exert additional cell adhesion effects, the authors demonstrated that promotion of migration was not associated with this role but with the establishment of functional gap junctional communication (GJC). Astrocytes -to which glioblastoma cells are phenotypically most closely related- are widely inter-connected through gap junctions, formed predominantly by the protein Cx43 [9] and glioma cells also express Cx43 [10-13].

Building up upon the concepts defined by Zhang et al. (1999) and Lin et al. (2002), we have explored their clinical relevance by looking at the ability of human glioblastoma cells to establish functional gap junctions with astrocytes, and at the role that such functional interaction may play in their migratory behavior. To observe and quantify the migration of human glioblastoma cells in a three-dimensional brain environment *ex vivo*, our analysis has taken advantage of *ISIS*, the "intra-slice implantation system" that we have recently developed [14], in addition to *in vitro *glioma cell cultures and glioma/astroglial co-cultures. The *ISIS *set-up allows the implantation not only of human tumor cells but also of fragments of biopsied glioblastoma into rodent brain slices maintained in culture [14,15]. It allows for long term analysis of glioma cell migration in a three-dimensional organotypic environment that mimics that of the brain parenchyma and application of pharmacological agents to disrupt GJC.

## Results

### Heterocellular coupling and migration of human glioblastoma cell lines

**Figure 1:**
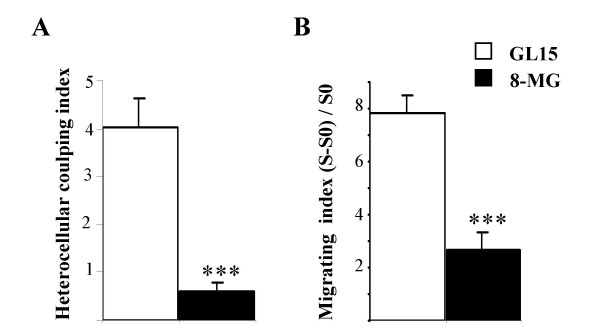
**Heterocellular coupling of glioma cell lines, and migration assay in brain slice cultures. **A, heterocellular coupling between astrocytes and human glioma cell lines (using the preloading method, see also fig. 6A). In contrast to 8-MG cells, GL15 cells established extensive GJC with normal astrocytes. Histograms represent mean heterocellular coupling indices (number of recipient astrocytes per double labeled tumor cell) of a minimum of three independent experiments (range 4 – 6; n= 120 to 225 donor cells per group; ± SEM; ***, p < 0.001). B, 8-MG cells and GL15 cells migration in brain slice cultures. Histograms of the average migration indices showing that GL15 were more invasive than 8-MG cells (mean values ± SEM. ***, p < 0.001).

Heterocellular coupling was assessed in glioma-astrocytes co-cultures, using the dual-label technique, in which glioma cells were pre-labeled donors cells and astrocytes potential recipient cells labeled through gap-junctional diffusion of the dye. Heterocellular coupling was more extensive for GL15 cells (heterocellular coupling astrocytes/glioma ratio: 4.05 ± 0.6 recipient astrocytes per one GL15 donor cell) than for 8-MG cells (0.6 ± 0.2; p < 0.001) (Fig. 1A).

Analysis of tumor cells invasion was assessed in brain slice cultures, in which both homocellular coupling (between glioma cells) and heterocellular coupling with astrocytes can occur. GL15 cells displayed a significantly higher invasive potential than 8-MG cells [(S - S_0_) / S_0 _= 7.82 ± 0.68 vs. 2.67 ± 0.67, p < 0.001] (Fig. 1B).

**Figure 2:**
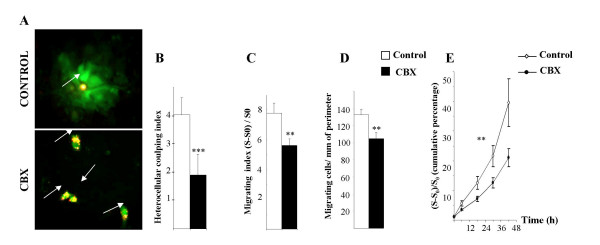
**Effects of inhibition of heterocellular coupling between GL15 cells and astrocytes**. A, Photomicrographs in co-cultures (preloading method) showing heterocellular coupling without (upper panel) or with addition of the inhibitor CBX. GL15 donor cells pre-labeled with a non-diffusing membrane-bound dye (DiI, *red*) and loaded with a fluorescent gap junction-permeable dye (calcein, *green*) were seeded on a monolayer of unlabeled astrocytes. Functional heterocellular GJC is visualized as the transfer of calcein from DiI-labeled donor cells (small arrows) to surrounding recipients cells. Donors cells appear *yellow *because of the merge of *red *and *green *labeling. CBX reduced the number of calcein-containing (*green*) recipient astrocytes, (×200). B, Histograms representing mean values of a minimum of three independent runs as in A (range 4 – 6; n = 160 to 225 donor cells per group ± SEM). (***, p < 0.001). C and D, histograms of the migration indices of GL15 in brain slices without or with CBX, illustrating decreased migration in the treated group (mean values ± SEM. **, p < 0.01). E, Cumulative migration indices plotted against time in co-cultures where GL15 cell spheroids were plated upon an astrocyte monolayer, without or with CBX; (S - S_0_) / S_0 _values were measured every 12 h, for 48 h. Integrated areas under the two curves in arbitrary units were compared, p < 0.0001.

Addition of CBX induced a significant decrease in the heterocellular coupling index of GL15 cells with astrocytes (down to 1.89 ± 0.74; p < 0.001) in co-cultures (Fig. 2B) whereas coupling was similar in control cultures either untreated, or treated with the functionally inactive analogue of CBX, GZA. Inhibition of GJC by CBX following implantation of GL15 into brain slices, resulted in a significant decrease in GL15 cells invasion into the brain parenchyma. The surface ratio (S-S_0_) / S_0 _of migration was reduced by inhibition of the gap junction function (5.59 ± 0.49 for CBX-treated vs. 7.82 ± 0.70 in untreated controls; p < 0.01) (Fig. 2C), as well as the number of GL15 cells that left the tumor mass (105 ± 6.3 cells/mm of perimeter vs. 132 ± 7.2; p < 0.01) (Fig. 2D). There was no difference between results obtained in the GZA and control groups. Those results were confirmed when migration of glioma cells out of spheroids was analyzed over an astrocyte monolayer culture in a 42 hours-long time lapse experiment (Fig. 2E).

In contrast, CBX treatment had no significant effect on 8-MG migration, either in glioma-astrocytes co-cultures or following implantation into brain slices (data not shown).

### Inhibition of GJC reduces invasion ability of cells either from human glioma xenografts maintained in nude mice or from fresh human glioma biopsies

The composite cellular content of glioma fragments and the technical difficulty in maintaining cells alive after seeding them in culture as cell suspensions, precluded analysis of the functional coupling of tumors, either maintained in nude mice or freshly biopsied. We, therefore, relied upon the effects of CBX to assess whether, similarly to results obtained in established glioma cell lines, inhibition of GJC may affect the migration of tumor cells out of a fragment and into the neural parenchyma of a brain slice.

Invasive capacities of the seven tumors maintained in nude mice were quite heterogeneous. Three were minimally invasive according to our criteria [total number of cells that detached from the tumor mass was less than 15 cells and the distance of migration less than 50 μm (T3, T6 and T8)]. These three tumors were precisely the ones in the series of 7 tumors to not exhibit any response to CBX treatment (Table 1). Among tumors characterized as "invasive" according to our criteria, CBX treatment significantly inhibited invasion in three (T2, T4 and T7), and had no statistically significant effect in one (T1). Inhibition concerned both the surface ratio of migration (35 to 50% decrease) and the number of migratory cells (25% to 40% decrease) (Table 1). Treatment with the inactive analogue GZA had no effect on migration as compared to controls.

**Table 1:** Cx43 expression and invasive capacity of gliomas maintained in nude mice

			***Controls***		***CBX***	
				
Tumor	Tumor^§^	Cx43	(S-S_0_)/S_0_	Cells/mm perimeter	(S-S_0_)/S_0_	Cells/mm perimeter
T1	TG-4-GEN	+++	1.15 ± 0.23	77 ± 9.4	1.05 ± 0.11	68 ± 5.6
T2	TG-1-HAM	+++	0.56 ± 0.05	81 ± 9.4	0.29 ± 0.08 ***	50 ± 7.5 *
T3	TG-7-ROM	+	0^a^	-	0^a^	-
T4	TG-14-RAV	+++	1.11 ± 0.08	73 ± 6.7	0.71 ± 0.04 **	54 ± 4.7 *
T6	TG-5-RAI	+/-	0^a^	-	0^a^	-
T7	TG-14-CHA	+++	0.45 ± 0.08	48 ± 4.2	0.22 ± 0.01 ***	35 ± 4.4 *
T8	TG-20-THO	+/-	0^a^	-	0^a^	-

^*a*^, cut off : a tumor which exhibited less than 15 migrating cells distance of migration, if any, less than 50 μm, was considered non invasive.

§ Xenograft tumor nomenclature as originally described by Leuraud et al (2003).

n = 7–10 per group and condition. ***, p < 0.001. **, p < 0.01, *, p < 0.05

**Figure 3:**
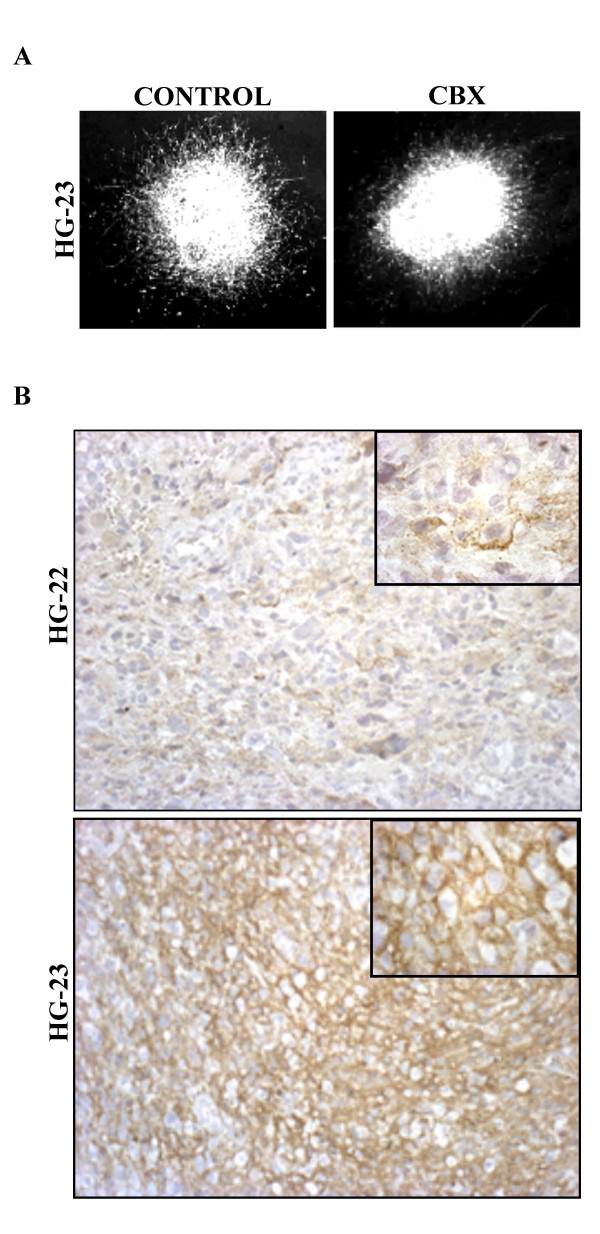
**Inhibition of gap junctional communication in biopsied human gliomas. **A. Vimentin immunostaining of the biopsied human glioblastoma HG-23 7 days after implantation into a cultured brain slice without (left panel) or with (right panel) CBX. Note the inhibition of cell migration when GJC is blocked. B, Connexin 43 immunocytochemistry (DAB, counterstained with hematoxylin) in two glioblastomas showing either moderate (HG-22) or abundant (HG-23) expression of the protein (×200). At the cellular level (inserts, ×1000), staining is dot-like or linear, predominantly at the membrane.

In order to test whether the results obtained with experimental gliomas could be extrapolated to native tumors, the migration potentials of biopsies from 4 human high grade gliomas (HG) were studied in brain slice culture, with or without addition of CBX or GZA. Overall, CBX treatment reduced migration indices of these four tumors by 30% (range 20 to 50%; p < 0.05 unpaired t test and Mann & Whitney – Table 2); this was the most pronounced for HG-23 (p < 0.02, Fig. 3A).

**Table 2:** Cx43 expression and invasive capacities of human biopsed gliomass

		***Controls***	***CBX***
			
Tumor	Cx43-IR	(S-S_0_)/S_0_	(S-S_0_)/S_0_
HG-18	+	4.89 ± 0.85 (n = 6)	3.63 ± 0.86 (n = 6)
HG-19	+	8.12 ± 0.83 (n = 9)	6.69 ± 0.88 (n = 7)
HG-22	+	3.64 ± 0.66 (n = 9)	2.92 ± 0.21 (n = 8)
HG-23	+++	5.48 ± 0.85 (n = 6)	2.92 ± 0.47 (n = 6)

IR: immunoreactivity

### Cx43 expression in glioma cells

In order to investigate whether Cx43 was instrumental in the establishment of functional coupling between glioma cells and astrocytes, Cx43 expression was analyzed in glioma cells from GBM cell lines and fresh human biopsies used in previous experiments. We chose to focus on Cx43 because it is the major Cx in astrocytes and is expressed by glioma cells [10-13].

**Figure 4:**
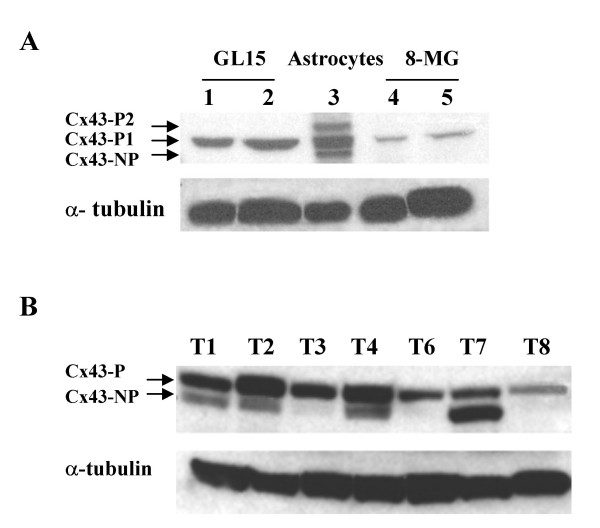
**Connexin 43 expression in glioma cells**. A, Western blot analysis of Cx43 expression in cell lines GL15 (lanes 1 and 2) and 8-MG (lanes 4 and 5), and in primary mouse astrocytes (lane 3). Three isoforms of Cx43 were detected in normal astrocytes, that corresponded to two phosphorylated (Cx43-P1 and Cx43-P2) and one non-phosphorylated (Cx43-NP) isoforms. GL15 cells exhibited higher level of Cx43 protein than 8 MG cells. B, Western blot analysis of Cx43 expression in seven human gliomas maintained in nude mice (T1 to T8). All tumors expressed one phosphorylated isoform. In addition, the non-phosphorylated isoform was detected in 4 tumor samples (T1, T2, T4 and T7). Tubulin was used as internal control The gels shown, are representative of a minimum of three independent experiments.

In agreement with previous studies [16], three isoforms of Cx43 could be separated on SDS-PAGE and visualized by immunoblotting as three distinct bands in control astroglial primary cultures: Cx43-NP, a non-phosphorylated isoform, Cx43-P1 and Cx43-P2, the two phosphorylated isoforms of the protein (Fig. 4). The Cx43-P1 isoform was prominent and Cx43-P2 and Cx43-NP isoforms were equally present (Fig. 4). As for invasive potential, Cx43 expression pattern was different in the two GBM cell lines with the GL15 cells exhibiting a three-fold higher concentration of the Cx43-P1 as compared to 8-MG cells (p < 0.001; Fig; 4A).

**Figure 5:**
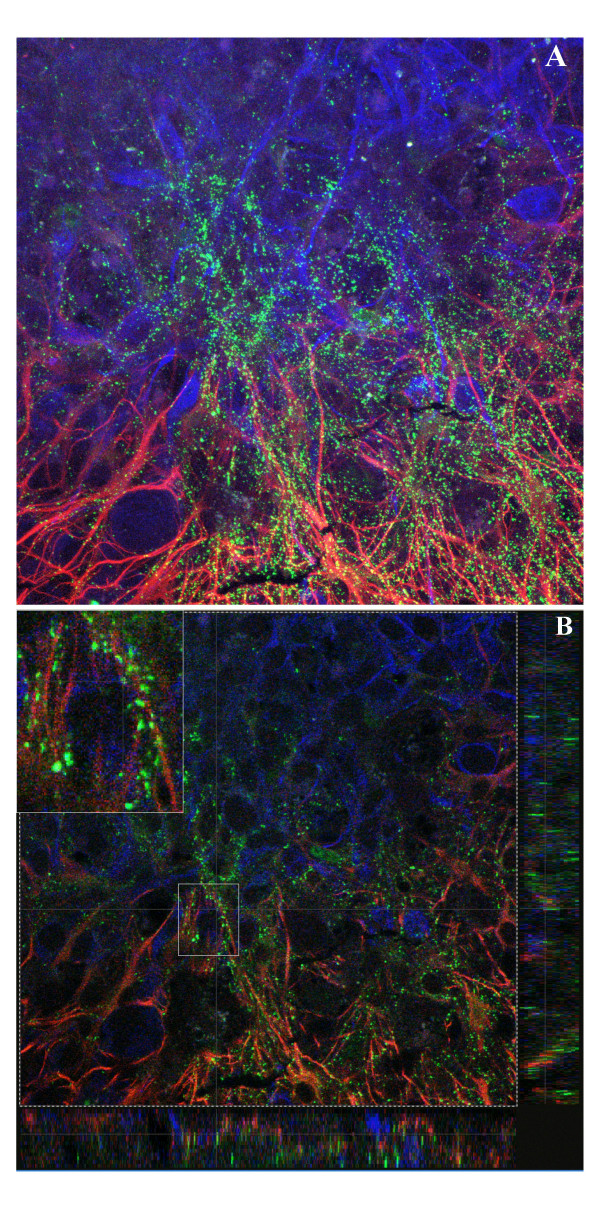
**Cx43 immunoreactivity in brain slice culture injected with GL15 glioma cells. **A, 3D projection of 15 consecutive confocal sections (0.5 μm) (×630), showing cooperation between migrating GL15 glioma cells (vimentin imunostaining, blue) with host astrocytes of the brain slice (GFAP immunostaining, red). Cx43 protein (green), is overexpressed by both tumor cells and astrocytes at the tumor margin where their processes are entwined. B, a single confocal section and two orthogonal projections (right and lower margins) corresponding to the overlayed perdendicular line. Enlarged view, of area boxed in B shows punctuate Cx43 immunoreactivity corresponding to gap junction plaques, at the interface of glioma cells and astrocytes strongly suggesting formation of heterocellular gap junctions. Note astrocyte morphologic changes which extend bipolar processes parallel to the long axes of the tumor cells

Histological analysis of Cx43 immunoreactivity in brain slices implanted with GL15 cells, showed that the protein was overexpressed in the tumor margin where glioma cells and astrocytes processes were intimately apposed and intermingled (Fig. 5). Confocal microscopy of brain slices allowed us to identify regions of enhanced immunostaining for Cx43 at the interface between GL15 cells and astrocytes, suggesting the existence of heterocellular gap junctions (Fig. 5). Interestingly, these astrocytes displayed a bipolar morphology that extended in arrays a long process that paralleled the outward migrating cells. This was not observed when 8-MG glioma cells were implanted.

A general expression of the P1 isoform was observed in the seven tumors maintained in nude mice, and a variable expression of the Cx43-NP isoform. The four invasive tumors (T1, T2, T4 and T7) expressed abundantly both Cx43 isoforms Cx43-NP and Cx43-P1, whereas the three others (T3, T6 and T8) which were not invasive and did not exhibit any response to CBX treatment, expressed lower levels of Cx43-P1 isoform and did not present detectable levels of Cx43-NP. (Fig. 4B). No expression of Cx43-P2 was recorded.

The amount of tissue available for experimental analysis did not allow us to carry out western blotting on freshly biopsied tumor fragments. Thus Cx43 expression in the human tumor biopsies was evaluated by immunohistochemistry on paraffin embedded tissue section.

Cx43 immunostaining was seen in all 4 specimens. Staining was patchy, and both the intensity of staining and the number of immunostained cells in patches differed from one sample to another. In the typical case, a positive microscopic field contained several strongly stained cells, and several unequivocally labeled cells of lower intensity (Fig. 3B). Homogenous fields of particularly strong immunolabeling intensity was observed in the HG-23 specimen.

### Homocellular coupling and migration of human glioblastoma cell lines

**Figure 6:**
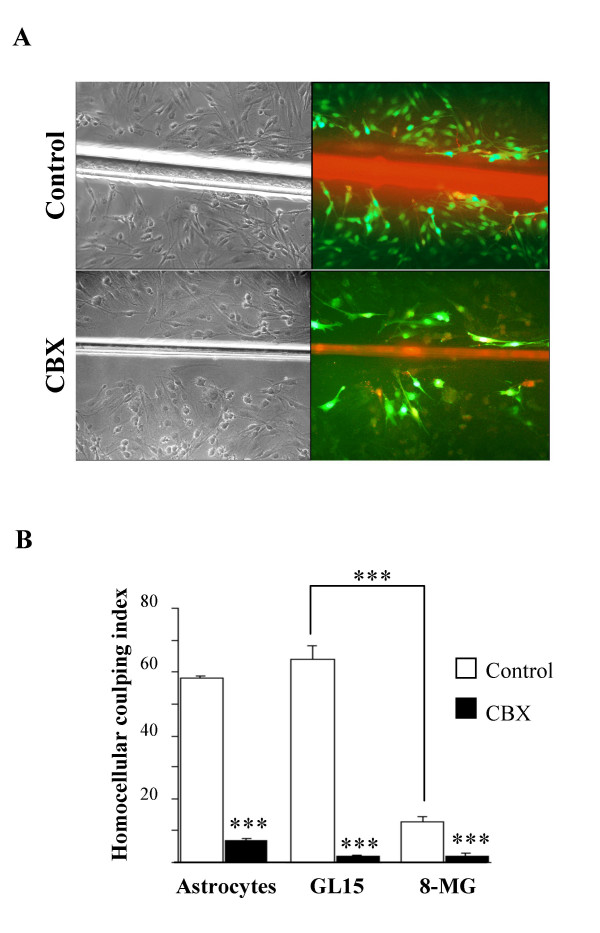
**Homocellular coupling in human glioma cell lines using the scrape loading method. **A, Dye coupling in absence (upper row) or presence (lower row) of the gap junctional blocker CBX. After scraping, injured cells were loaded with Lucifer Yellow (LY) and Rhodamin Dextran, and GJC was assessed by the number of neighboring cells that secondarily acquired green fluorescence. *Left panel*, confluent monolayer of GL15 cells in phase contrast. *Right panel*, centrifugal (left to right) diffusion of the dye tracer LY (*green*) from initially loaded injured cells (*yellow*, because of the merge of *red *and *green*) in the same monolayer. (×100). B, histogram representing the homocellular coupling indices (mean values ± SEM) from 4 to 6 independent cultures for each condition and cell type (astrocytes, GL15 or 8-MG). Homocellular coupling index = % labeled cells out of a total of ~400–800 analyzed cells per cell culture. Addition of CBX resulted in a dramatic decrease of the homocoupling in all cases ***, p < 0.001.

We have next evaluated homocellular coupling between glioma cells by the scrape loading method, in which homocellular coupling is demonstrated by the diffusion of the dye from mechanically lesioned cells to their neighbors in a monolayer culture. Assay revealed extensive coupling of GL15 (homocellular coupling index: 63.75 ± 4.4 %), comparable to that displayed by astrocytes in parallel control experiments (60 ± 5.2%), whereas 8-MG cells were only weakly coupled (12.7 ± 1.87 %; p < 0.001 vs. GL15), a result in keeping with their low level of Cx43 expression. Homocellular coupling of all cell types was strongly affected by addition of the gap junctional blocker CBX: down to 7.4 ± 1.3 % for astrocytes (p < 0.001), to 1.97 ± 0.47 for GL15 (p < 0.001) and to 2.2 ± 0.47 for 8-MG cells (p < 0.001) (Fig. 6), whereas GZA had no effect.

**Figure 7:**
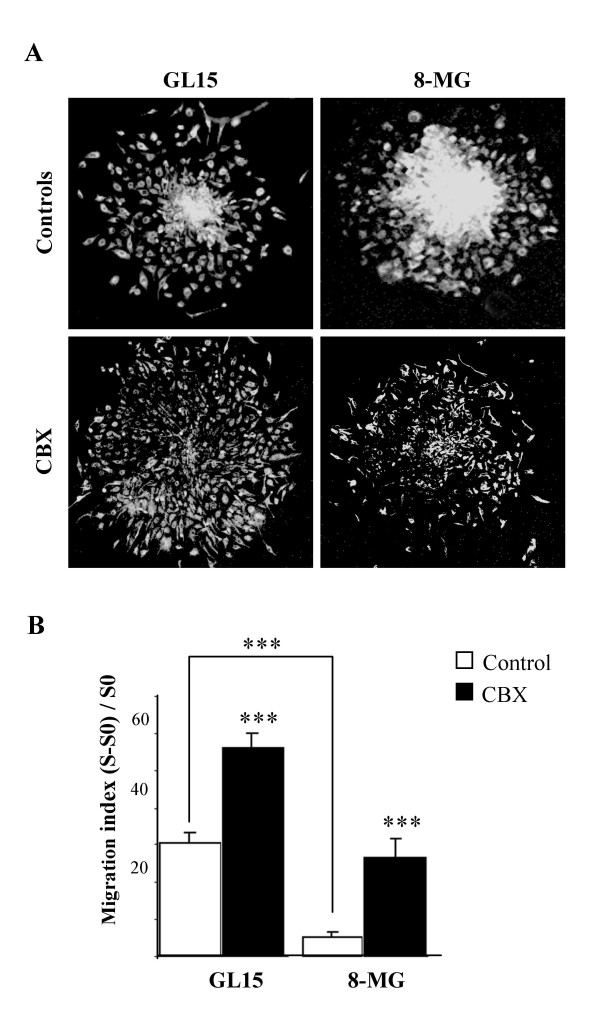
**Migration assay of glioma cell lines on collagen IV. **A, vimentin-immunostained GL15 and 8-MG spheroids, after 4 days of culture on collagen IV without (upper row) and with (lower row) addition of CBX, (x50). B, (S - S_0_) / S_0 _ratios (mean values ± SEM) obtained from 10 to 30 spheroids per group and condition. Addition of CBX enhanced spheroid disintegration and centrifugal dispersion of migrating cells. ***, p < 0.001.

To ensure that CBX-induced decreased migration of GL15 cells was mediated by the effect of the compound on gap junction communication, rather than more generally on the phenotype of tumor cells, analysis of tumor cell migration was performed in experimental conditions in which only homocellular coupling could exist, i.e. after seeding of glioma spheroids on a collagen IV matrix. Surprisingly, when homocellular coupling between tumor cells was inhibited by CBX, both tumor cell lines displayed a significant increase in migration out of the spheroids [(S-S_0_) / S_0 _= 55 ± 4.1 and 25.4 ± 5.25 for GL15 and 8 MG, respectively, p < 0.001 vs. untreated in both cases] (Fig 7).

### Induction of glioma cell migration *in vivo *by overexpression of Cx43

**Figure 8:**
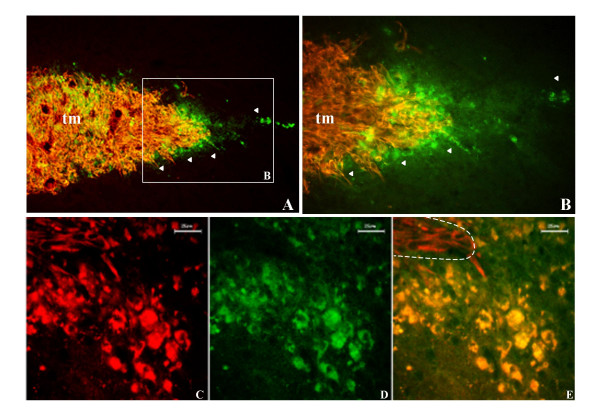
***In vivo *parenchymal invasion of C6 glioma cells overexpressing the fusion protein GFP-Cx43. **A, vimentin-immunolabeled C6 glioma cells (*red*) 8 days after striatal injection in the nude mice brain. C6 cells overexpressing the Cx43-GFP protein in green (arrow heads), are preferentially located at the tumor margin and display an invasive phenotype (×100). B, Microscopic field of the area boxed in A, at a higher magnificence (×200). C, D, confocal section (1 μm) of the border of another tumor showing invasive C6-Cx43-GFP cells at the tumor periphery migrating out. C6 cells which did not express Cx43-GFP protein, only labeled in *red *for vimentin remain in the tumor mass. E, is the overlay of the two channels. Migrating C6-Cx43-GFP cells appear *yellow *because of the merge of *red *and *green*. Dashed lines indicate the extremity of the tumor mass. Scale bar, 25 μm. tm, tumor mass.

In order to validate further the hypothesis of a role of GJC formed by Cx43 in tumor cell migration, we have used a technique adapted from that used by Lin et al. (2002) [8]. C6 tumor cells have been genetically engineered by transfection, using a plasmid containing a fusion gene encoding GFP attached to the carboxyl terminus of Cx43. The gap junctions formed by this chimeric protein were dye-permeable [17]. C6 cells are known to proliferate without migrating out of the tumor mass, following implantation into the rat brain, and do not express a significant amount of Cx43. By transplanting mixed population of native and GFP-Cx43-transfected cells, we explored the capacity for the latter to display specifically a migratory behavior. Indeed, GFP-Cx43 expressing C6 cells were preferentially located at the periphery of the tumor mass and some were even observed scattered in the surrounding brain parenchyma, strongly suggesting a migratory behavior (Fig. 8). This was not observed with C6-GFP cells which were, as classically described, only present in the tumor mass.

## Discussion

The main result of this study is the demonstration that functional GJC between tumor cells and host astrocytes is instrumental in the invasion of the brain parenchyma by human glioblastoma cells. Establishment of functional coupling between those two cell populations may be mediated by the gap junction protein Cx43 as ability of glioblastoma cells of various origins to migrate out of the tumor bulk into the brain parenchyma, appeared related to the level of Cx43 expression. In support of this hypothesis, overexpression of a fusion Cx43-GFP gene construct triggered a migratory behavior in otherwise non-infiltrative C6 glioma cells implanted into the brain. The similarities between those results and mechanisms of neural precursor cell migration during embryonic development suggest that glioma cells may subvert and take advantage of a physiological mechanism of cell migration into the brain. In contrast, homocellular interaction between glioma cells through gap junctions hampers their dissemination out of the tumor mass, pointing to an additional role for gap junctions, in cell-to-cell adhesion.

Over the past decade, studies on GJC in gliomas have mainly addressed its role in uncontrolled cell proliferation [18-20], by analogy with epithelial cancers in which a low level of connexin expression is a sign of profoundly perturbed tissue homeostasis and malignant evolution [21]. The notion that, in gliomas, GJC may also favor migration draws scientific argumentation from embryology and from observations that other neurectodermal tumors such as melanoma need to establish GJC with host cells in order to migrate out [6]. This concept has been strongly supported recently by the demonstration that a C6 rodent glioma cell line that had been genetically engineered to express Cx43 displayed a migratory behavior following transplantation into the rat brain [8]. This phenomenon was the more striking that the value of the rodent C6 cell line as an experimental model for glioblastoma has always been questioned because it did not reproduce the infiltrative phenotype which is a major characteristic of the human brain tumor. The induction of C6 migration was related to the establishment of functional GJC between glioma cells and host astrocytes through homotypic Cx43-Cx43 gap junctions.

Several lines of evidence drawn from our results converge to indicate that this concept is clinically relevant, as GJC with astrocytes also plays a role in the migration of brain tumor cells for human glioblastomas. Experimental results obtained here with native human glioblastoma cells of various origins, can indeed be interpreted as a demonstration of a similar role for GJC. First, pharmacological blockade of GJC by the specific inhibitor CBX [22] had negative effects on tumor cell migration, whereas its inactive analogue GZA [23] did not. Second, the gap junction protein Cx43, which is overexpressed by reactive astrocytes in the margin of glioblastomas [10], was also expressed by all studied human tumors, including biopsy specimens, providing a molecular basis for the potential formation of homotypic heterocellular gap junctions. Last, close membrane appositions at regions of reinforcement of Cx43 immunostaining, suggesting intercellular contacts between astrocytes and glioma cells migrating out of a tumor spheroid or the body mass, were observed in co-cultures of glioma cells on an astroglial monolayer, as well as following implantation of tumors into brain slices maintained in organotypic cultures.

The pharmacological approach used here had both advantages and drawbacks, which should be indicated as they may limit the conclusions to be drawn. The main advantage of that technique is the fact that it allowed us to study GJC inhibition in fresh tumor samples, in which biological properties and diversity of glioblastoma cells are well preserved, in contrast to the clonal selection and genetic variation that are commonly observed with cells lines. Conversely, we probably introduced some biological variability in the experimental system. This may be relevant to the fact that GJC inhibition with CBX did not abolish but rather reduced the rate of human glioma cell migration in our study, whereas Lin et al. (2002) obtained an "all-or-nothing" effect following genetic engineering of the C6 line. The partial effect on migration observed here may be due to the partial pharmacological inhibition consistent with the results of other studies [24,25] that demonstrated that CBX only reduced GJC by 50%. Alternatively, glioblastoma cell migration into the brain parenchyma does not relate exclusively to cell-to-cell interaction with astrocytes. Interactions between tumor cell and matrix components (reviewed by Giese et al., 2003) [5] are also major actors in glioma cell migration, and they are most likely not effected by inhibition of GJC, at least not directly. The use of a pharmacological compound also leads to questions about specificity. Various compounds used to block GJC have additional non-specific and toxic effects, among which halotane, octanol or acidifying agents. CBX has been shown to be nontoxic and has no effect on cell proliferation, total protein synthesis or cell viability [23,26,27]. This is ascribed, by some authors, to its mode of action, through direct binding on connexon particles [22]. We have checked the specificity of the GJC effects via different experimental ways, including a systematic negative control with glycyrrhyzic acid (GZA), the CBX precursor which only differs from it by its inability to disrupt GJC. CBX and its inactive analog GZA are known to both interact with mineralocorticoïd and glucocorticoïd receptors (see discussion in [28]), but these receptors do not mediate CBX-decreased GJC [23]. We also tested CBX effect on cell apoptosis, cell proliferation or cell viability in our conditions. No significant effect was observed. Although it has been reported that Cx43 may induce cell growth [18,29], we did not observe any effect of CBX on cell proliferation. It is interesting to mention that CBX-decreased GJC is mainly due to alteration of gap junction channel permeability, whereas Cx overexpression is sufficient to regulate cell growth [20,30]. As an interesting positive control of the specificity of the effects recorded in co-cultures and following implantation of tumor cells into brain slices, we have observed an opposite effect of CBX on cell migration when only homocellular interaction between glioblastoma cells was concerned (on collagen IV). Altogether, these results support the conclusion that non-specific pharmacological effects, if any, did not alter results of our study in a consequential manner.

Levels of Cx43 expression were positively related to glioma invasiveness. Analysis of Cx43 expression by western blot allowed detection of two distinct isoforms of the Cx43 protein, Cx43-P and Cx43-NP. Both isoforms were detected in the four highly invasive tumors (T1, T2, T4, T7), whereas the three minimally invasive tumors (T3, T6, T8) displayed no detectable levels of Cx43-P isoform. The presence of both Cx43 isoforms strongly correlated with the invasive capacities of the tumor. However, whether the migratory capacity may depend on the overall levels of expression, and/or upon the concomitant presence of both Cx43 isoforms is, at this stage, a matter of speculation. Published data on the association of either isoform with the functional state of gap junctions are conflicting. Phosphorylation of Cx43 either on serine or tyrosine residues has been reported to disrupt GJC (for review see [31,32]). This could in part explain why T3, T6 and T8 tumors, which only displayed the phosphorylated isoform -the inactive isoform of Cx43-, were non-invasive tumors. However, the reverse association between Cx43 dephosphorylation and GJC inactivation has been suggested in astrocytes [33]. Further biochemical analysis of Cx43 isoforms function in these cells may reveal whether the pattern of Cx43 isoform expression is involved in the invasive process and how this pattern can be altered by specific phosphorylation. On the other hand, it cannot be excluded that the abundance of both -P and -NP isoforms in migrating tumors simply relates to a dynamic turnover of connexons between the cytoplasm (Cx43-NP) and the cell membrane of a migrating cell, where Cx43-P may participate in short-lived contacts with host astrocytes [21]. Recent data have shown that CBX itself did not have any effect on the phosphorylation pattern of Cx43 [34].

In the biopsy specimens, levels of Cx43 expression were different from one tumor to another, and from one zone to another within a specimen, in agreement with results reported by other authors [10-13]. Interestingly, the highest expression of Cx43 was seen in HG-23, the tumor in which migration was the most substantially inhibited by CBX. In addition, transfection of C6 glioma cells with a Cx43 protein fused to a green fluorescent protein allowed us to trace C6-Cx43 cells after intrastriatal stereotactic implantation. Consistent with results of Lin et al (2002) and [35], only C6-Cx43 cells switched to an invasive phenotype providing a direct evidence of the positive correlation between glioma cells Cx43 expression and invasive potential. We hypothesize that Cx43 may favor glioma cells migration by facilitating glioma cells to form gap junction interactions with host astrocytes that will allow them, through currently unknown molecular mechanisms, to drag out from the tumor mass.

A peculiar morphological finding in this study was the phenotypic alteration of astroglial processes around tumors. Changes in the astroglial phenotype induced by glioma cells through GJC have been observed by other authors [7]. The *ISIS *protocol used here allowed us to gain a comprehensive three-dimensional view of the astroglial meshwork, and to identify the radial orientation of astrocytic processes toward the body of the tumors. This organization is reminiscent of the scaffolding of radial glial cells observed in the developing neocortex *in vivo *[36]and *in vitro *[37]. Radial glial cells extending long processes from the ventricular zone to the cortical plate [36]communicate with migrating neurons through Cx43 gap junctions [38-40]. Although tentatively, we may therefore speculate that astroglial Cx43 provide similarly support and guidance to the migrating tumor cells. In the same way as immature neuronal cells do [38,39,41], glioma cells may trigger the phenotypic change of surrounding astrocytes into radial glia-like cells, in order to make them suitable as a substrate for migration.

The three different migration assays allowed us to explore the importance of intercellular interactions between migrating cells and cellular environment occurring during invasion. We have shown that inhibition of homocellular GJC in GL15 spheroids seeded on collagen IV (i.e. in the absence of astrocytes), boosted glioma cell motility, resulting in a higher centrifugal dispersion of cells. This result is in keeping with that obtained by Lin et al (2002) in an experiment in which Cx43 was shown to mediate glioma cell adherence and aggregation. However, accelerated motility of GL15 glioma cells when CBX blocked homocellular glioma/glioma GJC was not observed when glioma cells additionally formed heterocellular GJC with astrocytes, *i.e. *on astrocyte monolayer or in brain slices. Quite the contrary, heterocellular glioma-astrocytes GJC seemed to be necessary for the invasive process to occur normally, as CBX significantly blocked it in those conditions.

## Conclusions

The purpose of our study was to determine whether the concept that functional gap junctional communication with host astrocytes facilitates invasion of malignant glioma cells put forward by other authors on the basis of experiments using a rodent glioma model (Lin et al, 2002), indeed apply to human glioblastoma and may, as a consequence, bear clinical relevance. This has indeed been clearly confirmed by the inhibitory effect of the specific GJC blocker, carbenoxolone, on the migration of human glioblastoma cells. Results obtained *ex vivo *on different supports of migration point to a functional difference between homocellular (glioma-glioma) and heterocellular (glioma-astrocytes) GJC. This may eventually be of prime therapeutic interest, by revealing mechanisms by which glioma cells disengage themselves from their neighbors in the tumor bulk, and establish new contacts with host astrocytes in order to migrate away [2,7].

## Methods

The role of GJC in human glioma invasion was investigated *in vitro *using three different types of preparations, namely glioblastoma cell lines, xenografts in nude mice, and freshly biopsied tumors. Because of selection for genotypes, the original cell heterogeneity of gliomas is restricted in xenografts and absent from cell lines. These models, therefore, do not fully reproduce the cell content of *in vivo *gliomas but, reciprocally, they present the advantage of being homogenous, easy to handle, and to allow analysis of large numbers of cells. *ISIS *uniquely permits to study, in addition, the invasiveness of freshly biopsied tumors with preserved cell repertoire. In those conditions, however, investigation is limited by the amount of available tissue.

### Human glioma cells

The two human glioblastoma (GB) cell lines used in the study, GL15 [42] and 8-MG [43] were grown as cell culture in glioma-cell medium: 50% minimum essential medium (MEM) and 50% Dubelco Modified Eagle's Medium (DMEM) complemented with 10% fetal calf serum (ATGC Biotechnological, France), glutamine 2 mM, D-glucose 3.3 mM, Penicillin 100 UI/mL, Streptomycin 100 μg/mL (all from Eurobio, France) at 37°C in a 5% CO2 humidified incubator. On confluence, cells were trypsinized, centrifuged at 300 G for 10 minutes and resuspended in 1 mL of glioma-cell medium. 5 × 10^5 ^cells were seeded in 1.5% agar-coated flasks (25 cm^3^, Falcon, France) for 7–10 days to obtain GL15 and 8-MG spheroids. Spheroids with a diameter ranging from 50–100 μm were selected for migration assays.

Seven grade III-IV human glioma biopsies xenografted and maintained subcutaneously in Swiss *Nu/Nu *mice (Charles River, France), kindly provided to us by Dr M-F. Poupon and P. Leuraud [44], were used in this study. For the sake of simplicity these tumors are referred to in the text as T1, T2, T3, T4, T6, T7 and T8 (see table 1). Mice were sacrificed when the tumor bulk reached a diameter larger than 1 cm. The tumors were retrieved and immediately transferred in the glioma-cell medium. Areas of necrosis and hemorrhage were identified under a microscope and discarded. The remaining tissue was cut into three fragments that were used as follows: -one was immediately implanted into a healthy nude mouse, in order to maintain the model; -a second one was immediately frozen for Western Blot analyses; -the third one was cut into smaller pieces for implantation into rodent brain slices maintained in organotypic cultures (see below).

Finally, four fresh human biopsy specimens were obtained from stereotactic or resection biopsies in the operating room. All samples were retrieved by the neurosurgeon from areas of enhanced contrast on magnetic resonance imaging, which correspond to a hypercellular zone on histological examination of the tumor. After retrieval, samples were immediately transferred in glioma-cell medium and prepared for implantation into brain slices. Pathological analysis of the same tumors was carried out in parallel on adjacent specimens; it systematically included immunohistochemistry for the connexin protein Cx43 in addition to usual stains. According to the WHO 2000 classification, these tumors were glioblastomas in three cases, an anaplastic grade III astrocytoma in one. For the sake of simplicity the generic term of glioblastoma will be used in the rest of the text.

### Functional assessment of gap junctional communication

Functional assessment of gap junctional communication (GJC) was performed on glioblastoma cell lines. Homocellular GJC was determined by the scrape-loading/dye transfer technique [45,46]. Briefly, either GL15, 8-MG or astrocytic cell cultures were incubated for 10 minutes in HEPES buffer solution (140 mM NaCl, 5.5 mM KCl, 1.8 mM CaCl_2_, 1 mM MgCl_2 _10 mM glucose and 10 mM HEPES pH 7.3). Cells were then washed in a Ca^2+^-free HEPES solution to prevent uncoupling. After scraping with the razor blade cells were incubated in the same solution containing 1 mg/ml of Lucifer Yellow (LY) (Sigma, France) and 1 mg/mL of Rhodamin Dextran 10,000 MV (Molecular Probes) for 1 minute. Dye transfer through GJC was assessed 10 minutes after scraping, using an Axioplan-2 microscope and appropriate filters (Carl Zeiss, Inc; Oberkochen, Germany). Fluorescent and bright light images digitized with a CoolSNAP camera (Ropper Scientific; Tuscon, AZ) from every microscopic field were merged, and the homocellular coupling index was calculated as the ratio of the number of LY-labeled cells to the total number of cells.

Heterocellular GJC was evaluated in glioma/astroglial cells co-cultures by a method adapted from Goldberg et al. (1995) [27]. Tumor cells were loaded for 20 min with a dual-label dye solution containing isotonic PBS-glucose (0.3M), 5 μM Calcein-AM and 10 μM DiI (both from Molecular Probes, France). They were then rinsed three times with isotonic PBS-glucose 0.3M, trypsinized and centrifuged. Five hundred Calcein/DiI-labeled cells (donor cells) were seeded on an unlabeled confluent astrocytic monolayer (recipient cells), and incubated at 37°C in a 5% CO2 humidified incubator for 20 h. Heterocellular coupling was calculated as the ratio of recipient cells (calcein-labeled astrocytes) to donor cells (Calcein/DiI-labeled glioblastoma cells). It is important to underline that this technique is different from the scrape loading method and uses another dye tracer to assess the gap junctional coupling. Hence, their results cannot be readily compared in quantitative terms.

Effects of inhibition of GJC were evaluated by treating the cultures with carbenoxolone (CBX); controls received its inactive analog, glycyrrhyzic acid (GZA) [23] (both from Sigma, France). CBX is a widely used specific inhibitor of GJC likely exerting its action by changing the conformation of gap-junction channels [22], while GZA has no effect on GJC. CBX and GZA treatments were carried out at a concentration of 30 μM. Cytotoxicity of these molecules on the two glioblastoma cell lines and on astrocytes had been tested in preliminary experiments. There was no significant increase in the lactate dehydrogenase medium/cell ratio for up to 7 days at the concentrations used (Cytotoxicity Detection Kit, Roche Molecular Biochemicals, France).

### Cell migration assays

Migration of glioblastoma cell lines was analyzed in three different experimental *in vitro *set ups: on collagen IV-coated cell culture dishes, after seeding on an astrocytic monolayer culture, and following implantation into organotypic brain slices (*ISIS*, Intra-Slice Implantation System). Migration out of tumor fragments issued from xenografted gliomas or from freshly biopsied specimens were only studied using the *ISIS *method.

Migration of GL15 and 8 MG cells out of spheroids deposited onto collagen-coated (0.5 μg/mL; Sigma, France) cell culture dishes was analyzed over 4 days.

For astrocytic co-cultures, primary astrocyte cultures were obtained from cerebral hemispheres of neonatal C57/bl6 mice (Charles River, France) as previously described [47]. Cells were grown in astrocyte medium: MEM (GIBCO, France) complemented with 10% FCS (ATGC Biotechnological, France), glutamine 2 mM, D-glucose 33 mM, Penicillin 100 UI/mL, Streptomycin 100 μg/mL and amino acids 0.5X (all from Eurobio, France). Medium was changed every 3 days. On confluence, GL15 and 8-MG spheroids were seeded on top of the astroglial monolayer, and cultures were continued for 4 days.

The *ISIS *migration assay was carried out as described previously [14], using brain slices obtained from postnatal day 6 C57/bl6 mice. Briefly, after decapitation the two hemispheres were separated and 400 μm-thick coronal slices were cut using a McIllwain tissue chopper (Mickle Laboratory, England). Slices were transferred into brain slices medium: MEM (GIBCO, France), 1 g/l D-glucose, 10% heat-inactivated fetal calf serum, 0.1 g/l transferrin, 16 μg/l putrescin, 40 μg/l N-selenium, 30 μg/l tri-iodothyronin, 5 mg/l insulin, and 60 μg/l progesterone (all from Sigma, France). Slices were separated and transferred onto Millicell-CM membranes (Millipore, France). The Millicell-CM membranes were kept in six-well plates, above 1 ml of brain slice medium. Slices were incubated at 37°C in a humid atmosphere with 5% CO_2_. The medium was changed three times a week. 24 hours after brain slice preparation, GL15 and 8-MG spheroids or small fragments of xenografted tumors and freshly biopsied specimens were deposited onto the slice surface and gently pushed with the tip of a needle, until the tumor tissue was well enveloped by the parenchyma of the slice. Cultures were maintained for 5 or 7 days for cell lines and tumor fragments, respectively.

In all experimental conditions, effects of the blockade of GJC were assessed using CBX (30 μM for collagen-coated and astrocytic co-cultures; 60 μM for brain slice cultures, a stronger dose because of the thinness of the slice), with two negative controls: untreated and GZA supplemented to the medium instead of CBX. These compounds were added to the culture medium 24 h after glioma spheroid or tumor implantation.

### *In vivo *experiments

For a control experiment, we transfected the C6 glioma cell line with a plasmid vector encoding either a green fluorescent protein (GFP) fused to the carboxyl terminus of Cx43 [17] kindly provided by Dr P. Martin, or else only GFP. C6-Cx43-GFP cells and C6-GFP control cells were implanted into the striatum of nude mice (5. 10^4 ^cells in 1 μL) (n = 5). Mice were sacrificed at day 8 and perfused with paraformaldehyde (4%) 8 days after implantation. Brains were sectioned serially on cryostat at 30 μm thickness as previously described [48].

### Immunoblotting and immunohistochemistry

Immunoblot analysis for Cx43 was performed on cells and tumor samples that were suspended in lysis buffer containing Tris base 50 mM, pH 7.4, NaCl 150 mM, Triton X-100 0.5%, EDTA 1 mM, protease inhibitor cocktail 0.5% (Sigma, St. Quentin, France) and triturated to homogeneity. Homogenates were centrifuged at 13,000 G for 10 or 30 minutes (for cell lines and tumor samples, respectively), and supernatants were aliquoted and stored at -80°C. Total protein content was determined by the Lowry method with bovine serum albumin as a standard. Total protein (30 μg) was boiled for 5 min after addition of 10% glycerol/5% mercaptoethanol and separated on SDS-PAGE 12.5% gels, transferred on PVDF membrane (Bio-RAD, France) under classical conditions [49] and immunoblotted overnight at 4°C, using a polyclonal anti-Cx43 antibody (1:500, Zymed, France). Bound primary antibody was detected with a horseradish peroxydase-coupled anti-rabbit antibody (1:5,000; Amersham Pharmacia Biotech, France). In preliminary positive control experiments, Cx43 was revealed as three bands of about 41, 43 and 45 kDa, representing three different isoforms in heart sample. The two higher molecular mass bands have been shown to correspond to phosphorylated forms of the protein, and the 41 kDa migrating species to the unphosphorylated form of the protein [16].

Immunohistochemistry was carried out on cell and slice cultures and brain slices from *in vivo *experiments following fixation using 4% paraformaldehyde in PBS at 4°C (30 min for mono- and co-cultures, 4 h for *ISIS*). For fluorescent immunostaining, cultures were rinsed several times in PBS, then incubated for 1 hour in PBS containing 10% normal horse or goat serum and 0.6% Triton X-100 (Sigma). They were incubated overnight at 4°C with monoclonal antibodies against vimentin (1/400, Neo Markers, France), or polyclonal antibodies against glial fibrillary acidic protein-GFAP (1/100, Dako) or Green Fluorescent Protein-GFP (1/200,. Molecular Probes). After 3 washes in PBS, they were then incubated with a PBS solution containing anti-rabbit or anti-mouse immunoglobulin conjugated either to fluorescein isothiocyanate-FITC, AMCA (1/400; Vector, France), or to cyamidine-Cy3 (1/1000; Jackson Immuno Research, France). For human biopsies, immunocytochemical detection of Cx43 was carried out on formalin-fixed, paraffin-embedded 5 μm sections. Antigen-retrieval consisted in heating slides immersed in citrate buffer (pH 6) in a microwave oven (3 × 5 min at 750 W). To block non-specific binding sites slides were incubated in PBS containing 10% BSA. A primary polyclonal antibody (Zymed) directed against the intracellular portion of the Cx43 molecule was applied (1:200) to sections overnight. Bound primary antibodies were detected by a Vector Elite ABC kit (Vector Laboratories, Burlingame, California) following the manufacturer's instructions and using DAB as the chromogen. Slides were lightly counterstained in Harris's hematoxylin. Negative controls had the primary antibodies omitted. A specimen of normal human cortex from epilepsy surgery showed diffuse staining of astrocytic cells accentuated at perivascular and superficial locations, corresponding to astrocytic endfeet and the glia limitans, respectively.

### Quantification and statistical analysis

Fluorescent microscopy on a Zeiss Axioplan2 microscope and image analysis using the KS.400 (3.0 version) software were performed to quantify invasion of glioma cells, according to techniques that have been detailed elsewhere [14]. Two invasion parameters were assessed, (i) the maximum area of invasion, given as the ratio (S-S_0_) / S_0_, where S is the maximum area containing migrating glioma cells and S_0 _is the initial tumoral mass area, this ratio compensating for the heterogeneous sizes of implanted spheroids and tumor fragments, and (ii) the number of invading cells (cells/mm of S_0 _perimeter). For collagen-coated migration assay and astrocytic co-cultures, only the maximum area of invasion was assessed. The statistical significance was evaluated using Mann & Whitney test and Student unpaired t test.

## Authors'contribution

ROconceived and designed the study, performed acquisition, analysis and interpretation of data and drafted the manuscript. CCconceived and designed the study, performed acquisition, analysis and interpretation of data and drafted the manuscript. JSGparticipated in the design of the study and carried out xenografts in nude mice. SdB participated in the design of the study. SP provided human biopsies tissue and diagnosis. LV participated in the design of the study. MT have been involved in revising the article critically for important intellectual content. MPconceived, designed and coordinated the study, drafted the manuscript and have given final approval of the version to be published. All authors read and approved the final manuscript.
